# ClC-7 Deficiency Impairs Tooth Development and Eruption

**DOI:** 10.1038/srep19971

**Published:** 2016-02-01

**Authors:** He Wang, Meng Pan, Jinwen Ni, Yanli Zhang, Yutao Zhang, Shan Gao, Jin Liu, Zhe Wang, Rong Zhang, Huiming He, Buling Wu, Xiaohong Duan

**Affiliations:** 1 State Key Laboratory of Military Stomatology, Department of Oral Biology, Clinic of Oral Rare and Genetic Diseases, School of Stomatology, The Fourth Military Medical University, 145 West Changle Road, Xi’an, 710032, People’s Republic of China; 2 Department of Stomatology, Nanfang Hospital, Guangzhou; College of Stomatology, Southern Medical University, Guangzhou, 510515, People’s Republic of China; 3 State Key Laboratory of Military Stomatology, Department of Prosthodontics, School of Stomatology, The Fourth Military Medical University, Xi’an, 710032, People’s Republic of China; 4 The Interdisciplinary Nanoscience Center (iNANO) and Department of Molecular Biology and Genetics, Aarhus University, 8000 Aarhus C, Denmark; Xiangya Stomatological Hospital, Central South University, Changsha 410078, China; 5 State Key Laboratory of Military Stomatology, Department of Endodontics, School of Stomatology, The Fourth Military Medical University, Xi’an, 710032, People’s Republic of China

## Abstract

*CLCN7* gene encodes the voltage gated chloride channel 7 (ClC-7) in humans. The mutations in *CLCN7* have been associated with osteopetrosis in connection to the abnormal osteoclasts functions. Previously, we found that some osteopetrosis patients with *CLCN7* mutations suffered from impacted teeth and root dysplasia. Here we set up two *in vivo* models under a normal or an osteoclast-poor environment to investigate how ClC-7 affects tooth development and tooth eruption. Firstly, chitosan-*Clcn7*-siRNA nanoparticles were injected around the first maxillary molar germ of newborn mice and caused the delay of tooth eruption and deformed tooth with root dysplasia. Secondly, E13.5 molar germs infected with *Clcn7* shRNA lentivirus were transplanted under the kidney capsule and presented the abnormal changes in dentin structure, periodontal tissue and cementum. All these teeth changes have been reported in the patients with *CLCN7* mutation. *In vitro* studies of ameloblasts, odontoblasts and dental follicle cells (DFCs) were conducted to explore the involved mechanism. We found that *Clcn7* deficiency affect the differentiation of these cells, as well as the interaction between DFCs and osteoclasts through RANKL/OPG pathway. We conclude that ClC-7 may affect tooth development by directly targeting tooth cells, and regulate tooth eruption through DFC mediated osteoclast pathway.

ClC-7 is encoded by *CLCN7* in humans and the mutations in the *CLCN7* gene cause two different types of osteopetrosis: autosomal dominant osteopetrosis type II (OMIM166600) and autosomal recessive osteopetrosis type IV (OMIM611490)^1^. Previously we identified two Chinese osteopetrosis patients with *CLCN7* gene mutations. Besides the identical bone phenotypes of osteopetrosis, the patients presented hypodontia, enamel dysplasia, malformed teeth, altered tooth eruption, and impacted teeth^2^. The tooth germs were able to form but did not erupt in *Clcn7*^−/−^ mice^3^. A spontaneous autosomal recessive osteopetrosis mouse model with the mutant allele in *Clcn7* locus displayed no tooth eruption or tooth root formation^4^. A recent study found no root was formed in the molars of 3-week old *ClC-7* mutant mice^5^.

ClC-7 is expressed in the ruffled border of osteoclasts and dysfunctions of the ClC-7 affect osteoclast-mediated extracellular acidification, resulting in disturbed dissolution of bone inorganic matrix^3^,^6^. As we know, osteoclasts play an important role in tooth formation and eruption^7^,^8^. It is possible that the dysplastic tooth and the impact of tooth germ in osteopetrosis patients or in *Clcn7*^−/−^ mice are caused by the dysfunctional osteoclasts.

ClC-7 has been also reported in other types of cells or tissues such as neurons^9^, gingival tissues^10^, bone tissues^11^,^12^, etc, suggesting that ClC-7 might have new functions in these cells or tissues. The locations of ClC-7 in ameloblast, odontoblast, dental papilla cells and dental follicle cells (DFCs)^13^,^14^ also raise one hypothesis, whether ClC-7 directly affect tooth development.

Here we set up two *in vivo* models with a normal and an osteoclast-poor environment to examine the effect of ClC-7 on the tooth development and eruption. In addition, we investigated the role of ClC-7 roles in the differentiation and biological functions of several tooth cells, such as ameloblasts, odontoblasts and DFCs.

## Results

### Local *Clcn7* silencing impairs tooth morphogenesis and tooth eruption

**Figure 1:**
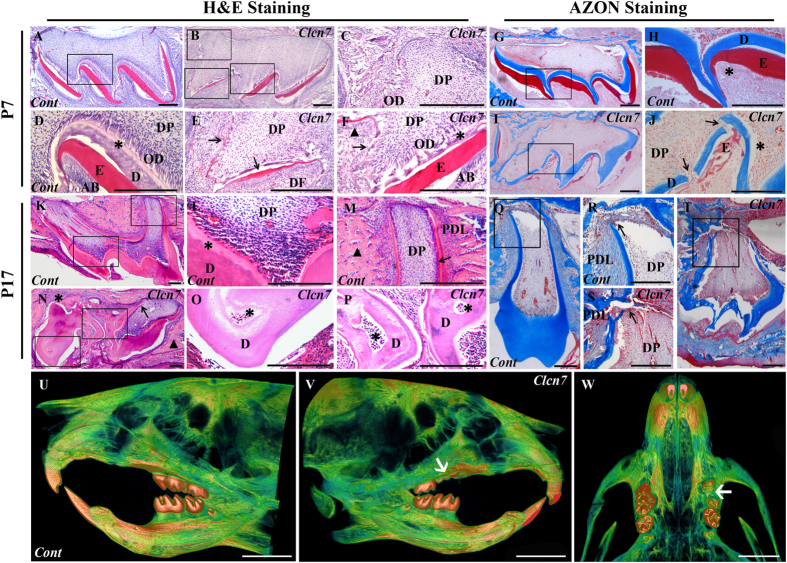
Histological and micro-CT characteristics of tooth germs treated with CS-siRNA particles. (**A–F,K~P**) H&E staining; (**G~J,Q~T**) AZON staining. The molars were harvested and sectioned at day 7 (P7) (**A~J**) and day 17 (P17) (**K~T**) from mice treated by control CS-siRNA or CS-*Clcn7*-siRNA. The P7 molar treated with CS-*Clcn7*-siRNA showed malformation of enamel and dentin (**B,C,E,F**). The integrity and continuity of enamel was interrupted (**E,F**) black arrow: enamel; black triangle: ameloblasts. (**I,J**). Dentin became disrupted and uneven (**J**, black arrow) and the boundary between dentin and predentin was indiscernible (**F**, asterisk), and the disorganized odontoblasts were found (**J**, asterisk). In P17 CS-*Clcn7*-siRNA group, the tooth was not fully erupted (**N,T**). The malformed tooth showed the abnormal cusps and short roots (**N**, black arrow; **T**) or disappeared root (**N**, asterisk). Abnormal and irregular fibrous tissues surrounded tooth instead of bone tissue (**N**, black triangle). The disorganized dentin and pulp cavity were observed (**O**,**P** asterisk). The shape of the tooth crown was irregular and the root dentin was disrupted (**S**, black arrow) and uneven. The continuity and integrity of cervical loop and Hertwig’s epithelial root sheath was disappeared (S, black arrow). Figure (**D,C,E,F,H,J,L,M,O,P,R,S**) are partial enlargement of figure (**A,B,G,I,K,N,Q,T**), respectively. *Cont* = CS-negative-siRNA treated tooth; *Clcn7* = CS-*Clcn7*-siRNA treated tooth. OD = odontoblasts; DP = dental pulp; DF = dental follicle; E = enamel; AB = ameloblasts; D = dentin; PDL = periodontal ligament. Scale bars = 200 μm. (**U~W**) Micro-CT scanning image of mouse head. U. Lingual view of the control side treated with CS-negative-siRNA nanoparticle. (**V**) Lingual view of the experimental side treated with CS-*Clcn7*-siRNA nanoparticle. W. Palatal view of the maxilla. The left first molar was impacted and lost its regular crown shape and cusps (white arrow). *Cont* = CS-negative-siRNA treated group; *Clcn7* = CS-*Clcn7*-siRNA treated group. Scale bars = 2 mm.

Comparing to the control (Fig. 1A,D,G,H), the enamel and dentin at day 7 post-injection became discontinuous and had uneven thickness in chitosan (CS)-*Clcn7*-siRNA group (Fig. 1B,C,E,F,I,J). On P17, the tooth with well developed crown and root was normally erupted in control group (Fig. 1K–M,Q,R); while CS-*Clcn7*-siRNA treated group showed abnormal dentin, irregular tooth roots (Fig. 1N,O,P,S,T) and impacted tooth (Fig. 1N,T). Micro-CT scanning showed that the left maxillary first molar treated with CS-*Clcn7*-siRNA nanoparticles became impacted and its eruption was delayed in P17 group (Fig. 1U~W).

### ClC-7 deficiency disrupts morphogenesis of tooth in kidney capsule

**Figure 2:**
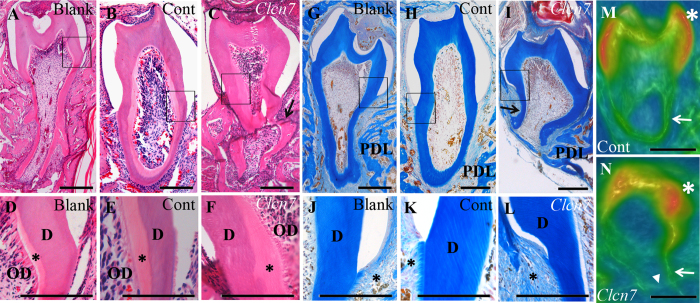
Histologic and microCT characteristics of transplanted tooth germs. H&E staining (**A–F**), AZON staining (**G–L**) and micro-CT scanning image (**M,N**) were taken from blank control (**A,D,G,J**), negative-shRNA group (**B,E,H,K,M**) and *Clcn7*-shRNA group (**C,F,I,L,N**) after 28 days of transplantation. In *Clcn7*-shRNA, the tooth roots became curved and lost the regular shape (**C,I**). The predentin was enlarged and the odontoblasts were irregular arranged (**F**, asterisk) comparing to the other two groups (**D,E**, asterisk). The periodontal tissue of *Clcn7*-shRNA group was filled with poor-arranged fibroblasts (**L**, asterisk) other than normal periodontal ligament, cementum and alveolar bone (**J,K**, asterisk). Crooked tooth root (**N**, arrow), abnormal cusps (**N**, asterisk) and enlarged apical foramen (**N**, triangle) were observed in micro-CT scanning image. Figure (**D–F,J–L**) were partial enlargement of figure (**A–C,G–I**), respectively. Blank = blank control group; Cont = negative-shRNA group; *Clcn7* = *Clcn7*-shRNA group. OD = odontoblasts; D = dentin; PDL = periodontal ligament. Scale bars = 200 μm.

After 3 days of lentivirus infection, obvious GFP fluorescence could be detected in the infected tooth germs, meanwhile, the *Clcn7* mRNA level was decreased about 70% in the *Clcn7*-shRNA treated group, compared to the negative-shRNA group (*P* = *0.0001*, data not shown). The teeth were well developed with clear profiles including normal cusps and roots in both blank control groups (Fig. 2A,D,G,J) and negative-shRNA group (Fig. 2B,E,H,K,M). The abnormal morphological alterations were found in *Clcn7*-shRNA group, such as short and/or crooked roots, abnormal cusps and apical foramen (Fig. 2C,I,N). Part of predentin became thicker and the odontoblasts lost their regular column shape (Fig. 2F). The imposing characteristics of AZON staining in *Clcn7*-shRNA group were demonstrated as the disorderly arranged periodontal tissues. Large amount of fibroblasts replaced the well-organized periodontal ligament, cementum and alveolar bone (Fig. 2L) compared with the control groups (Fig. 2J,K).

### Impact of ClC-7 on the expression of enamel and dentin matrix

**Figure 3:**
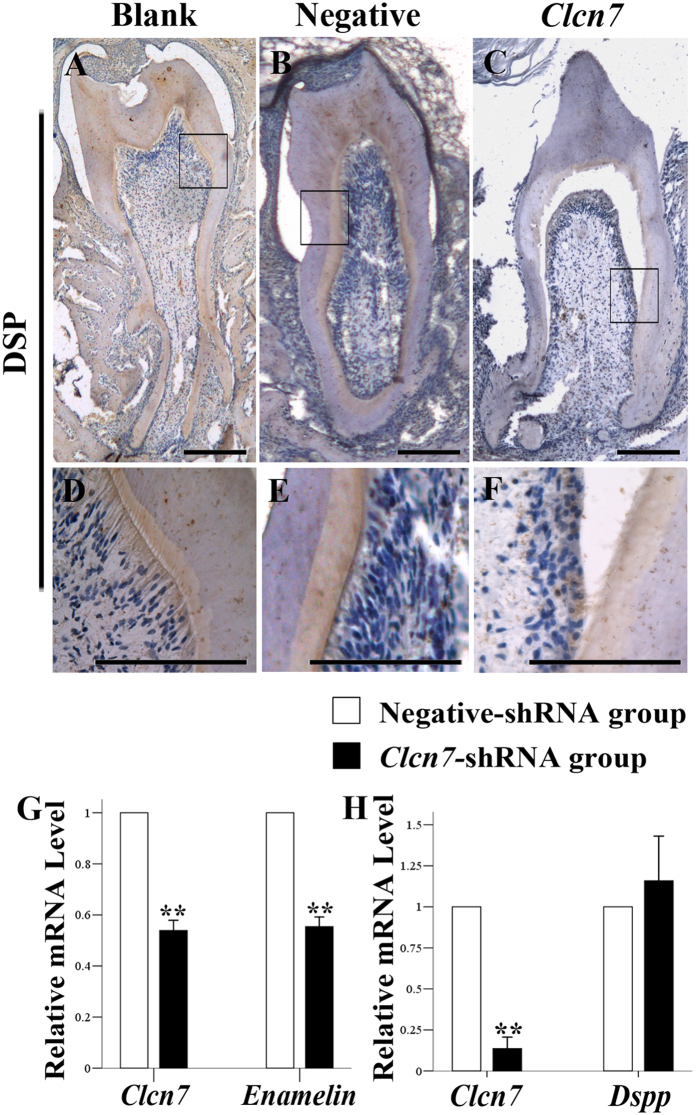
ClC-7’s effect on DSP and *Enam* expression. (**A–F**) Immunohistochemistry staining of DSP in the transplanted tooth. DSP was expressed in the pre-dentin, dentin and the odontoblasts (**A~E**). In the *Clcn7*-shRNA group, the expression of DSP was reduced (**C,F**) compared to the other two groups. (**D–F**) were partial enlargement of figure (**A–C**), respectively. Blank = blank control group (**A,D**); Cont = negative-shRNA group (**B,E**); *Clcn7* = *Clcn7*-shRNA group (**C,F**). Scale bars = 200 μm. (**G**) Real time PCR results in ameloblast LS8 cell line. Compared to the negative-shRNA group (RQ equals one), the RQ values in the *Clcn7-*shRNA group of *Clcn7* and *Enam* are 0.539 (0.069, *P* = *0.007*) and 0.554 (0.065, *P* = *0.007*), respectively. (**H**) Realtime PCR results of odontoblast MDPC23 cell line. Compared to the negative siRNA group, the RQ values in the *Clcn7-*siRNA group of *Clcn7* and *Dspp* were 0.137 (0.069, *P* = *0.002*) and 1.159 (0.273, *P* = 0.419), respectively.

The expression level of DSP was decreased in the group under the treatment of *Clcn7*-shRNA lentivirus infection (Fig. 3A~F). After lentivirus infection or siRNA transfection, the expression of *Clcn7* in ameloblast LS8 and odontoblast-like MDPC-23 cells was downregulated. Subsequently the expression level of *Enam* was decreased in LS8 cell line (Fig. 3G), while *Dspp* was not obviously changed in MDPC-23 cells (Fig. 3H).

### Impact of ClC-7 on the differentiation of DFCs

We confirmed the general mesenchymal characteristics of DFCs by positive immunocytochemical staining for anti-vimentin and negative staining for anti-pan cytokeratin (Supplementary Fig. 1).

**Figure 4:**
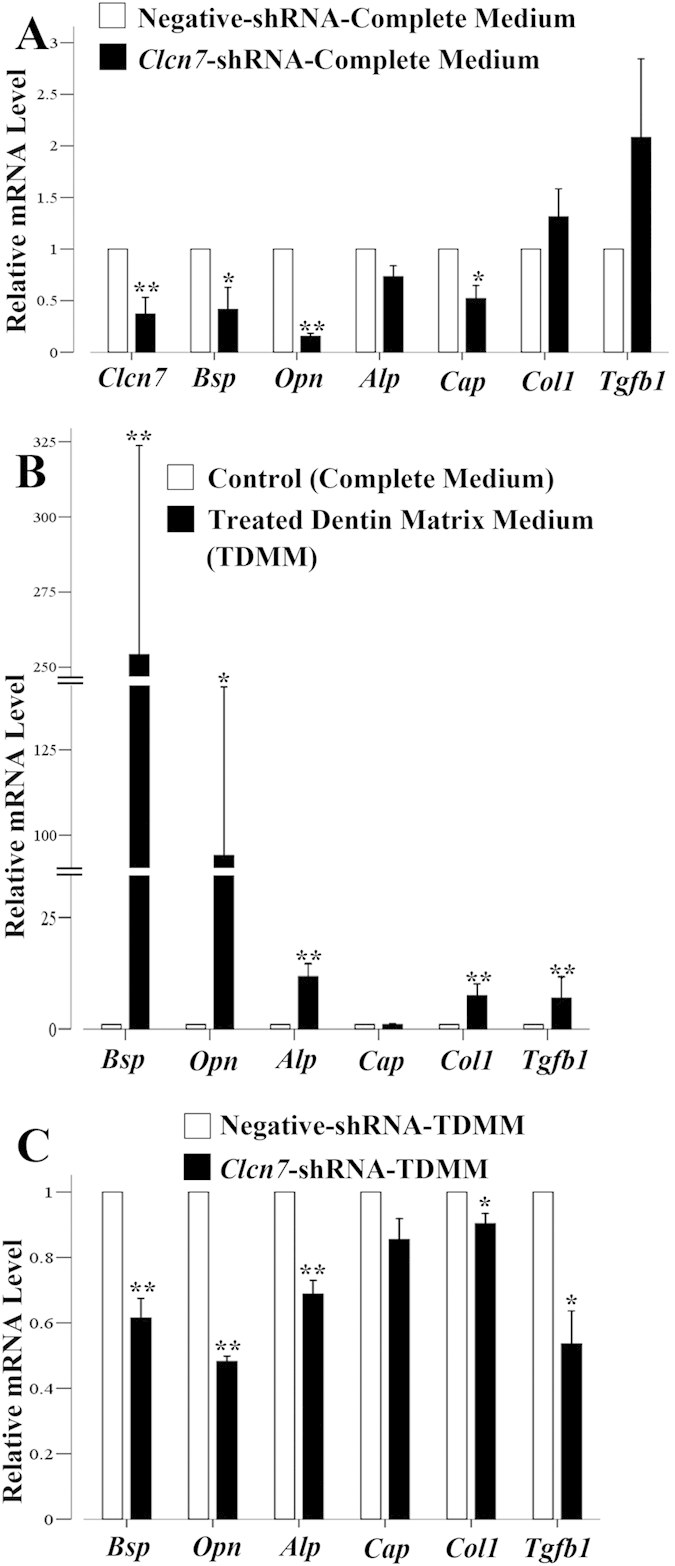
ClC-7’s effect on the expression of different marker genes of DFCs. (**A**) Real time PCR results with *Clcn7* or negative control shRNA lentivirus treatment. The RQ values in the *Clcn7-*shRNA group, which represents the mRNA level, of *Clcn7, Bsp, Opn, Alp, Cap, Col1, Tgfb1* are 0.370 (0.161, *P* = *0.000*), 0.416 (0.214, *P* = *0.042*), 0.154 (0.031, *P* = *0.017*), 0.733 (0.103, *P* = *0.046*), 0.521 (0.126, *P* = *0.022*), 1.313 (0.272, *P* = *0.351*), 2.082 (0.760, *P* = *0.132*), respectively, compared to the negative*-*shRNA group. B. Realtime PCR results of osteoblast-/cementoblast-related genes after 3 days’ treatment of TDMM. The RQ values of *Bsp, Opn, Alp, Cap, Col1, Tgfb1* in the TDMM group, are 254.23 (69.484, *P* = *0.001*), 94.074 (49.338, *P* = *0.033*), 11.706 (2.898, *P* = *0.005*), 0.978 (0.146, *P* = *0.819*), 7.427 (2.672, *P* = *0.006*), 6.855 (4.825, *P* = *0.007*), respectively, compared to complete medium control group. (**C**). Realtime PCR results of osteoblast-/cementoblast-related genes after 3 days’ TDMM treatment as well as lentivirus treatment. The RQ values of *Bsp, Opn, Alp, Cap, Col1, Tgfb1* in the *Clcn7*-shRNA group, are 0.615 (0.06, *P* = *0.008*), 0.482 (0.017, *P* = *0.000*), 0.688(0.042, *P* = *0.006*), 0.855 (0.063, *P* = *0.058*), 0.903 (0.031, *P* = *0.033*), 0.536 (0.101, *P* = *0.015*), respectively, compared to the negative-shRNA group. **P < *0.01*, *P < *0.05*. Data are presented as mean (SD, P value).

The osteogenic induction with treated dentin matrix medium (TDMM) upregulated the mRNA expression of *Alp, Bsp, Opn, Col1,* and *Tgfb1* (*P *< *0.01*) with various ratios; while these induced upregulations of *Alp, Bsp, Opn, and Tgfb1* were inhibited after *Clcn7* gene level was lowered by *Clcn7* shRNA lentivirus infection (Fig. 4A~C). However, the lowered *Clcn7* level did not have any effect on calcified node formation with alizarin red staining (Supplementary Fig. 2A~H).

### Effect of ClC-7 on osteoclast formation through DFCs

**Figure 5:**
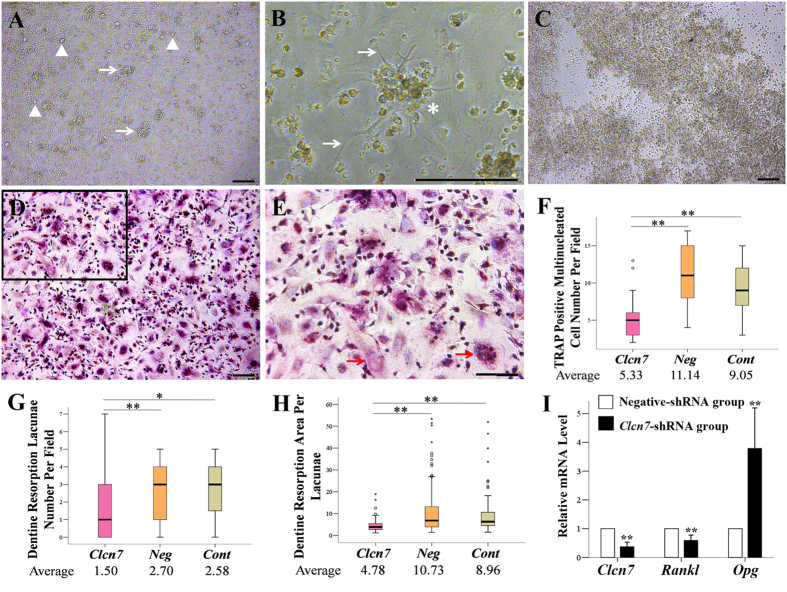
ClC-7’s effect on the role of DFCs mediated osteoclastogenic differentiation. (**A**) Phase contrast images of DFCs and MNCs co-cultured for 7 days at low magnification (**A**) and high magnification (**B**), white triangles in A represented DFCs, white arrows in A represented MNCs gathering round the DFCs and white arrows in B represent cellular process of DFCs. (**C**) Phase contrast image of LS8 cells and MNCs after 3 days co-culture. (**D**) TRAP-positive cells on day 7 of DFCs/MNCs co-culture at low magnification. (**E**) Magnified region from D. Red arrows represent TRAP-positive multinucleated cells. (**F**) Comparison of the number of TRAP-positive multinucleated cell number per field vision. The *Clcn7-*shRNA group showed less TRAP-positive mulitinucleated staining cells than that of the negative-shRNA group and the control group (*P* = *0.001*). (**G,H**) Dentine resorption area per lacunae (μm^2^, n = 50). The dentin resorption lacunae formed by osteoclast-like cells in the *Clcn7-*shRNA group are reduced in amount and became smaller in area, when compared with the negative-shRNA group (*P*_*number*_ = *0.003, P*_*area*_ = *0.000*) and the control group (*P*_*number*_ = *0.011, P*_*area*_ = *0.000*). Bold horizontal bars indicate average values. The circle represents the outliers and the small asterisk represents the extreme values. **P < *0.01*, *P < *0.05. Clcn7* = *Clcn7*-shRNA group; *Neg* = Negative-shRNA group; *Cont* = Control group. Scale bars (**A–E**) = 200 μm. (**I**) Realtime PCR results of *Opg* and *Rankl* after knocking down *Clcn7* in DFCs. The RQ value of *Opg* was upregulated to 3.789 (1.406, *P* = *0.000*) and *Rankl* downregulated to 0.589 (0.185, *P* = *0.008*), respectively. Data are presented as mean (SD, P value).

DFCs became round and lucent, and looked like multi-dendrites cells under the condition of DFCs/MNCs co-culture (Fig. 5A,B). The monocytes survived no more than three days in LS8 cells/MNCs co-culture system (Fig. 5C), but formed TRAP-positive mulitinucleated cells at day 7 post DFCs/MNCs co-culture, and lowered ClC-7 expression level resulted in less TRAP-positive mulitinucleated staining cells (Fig. 5D~F). These TRAP+ cells showed small and shallow pits on the dentin slices, and the numbers and area of resorption lacunae were decreased in ClC-7 deficiency group (Fig. 5G~H, Supplementary Fig. 3). qRT-PCR demonstrated the upregulation of *Opg* and downregulation of *Rankl* after *Clcn7* gene was silenced in DFCs (Fig. 5I).

## Discussion

Resorption of alveolar bones by osteoclasts is essential for tooth eruption^7^. The osteopetrosis patients caused by osteoclasts dysfunction usually presented impacted and malformed teeth and the involved genes include *RANKL*^15^,^16^, *TCIRG1*^17^, etc. ClC-7 is highly expressed in the ruffled membrane of osteoclasts, and responsible for acidifying resorption lacuna. Osteoclasts isolated from ClC-7 deficient mice failed to resorb calcified bone^6^,^18^, which might contribute to the tooth malformations and eruption abnormalities in osteopetrosis patients with *CLCN7* mutation^2^ and *Clcn7* gene knockout mice^3^,^5^.

In the present study, we formulated siRNA with chitosan to protect siRNA from degradation, extended its *in vivo* effects and mimicked the gene knockout effect of *Clcn7* around tooth germs^19^. We chose the first upper molar of P1 mouse as the siRNA target site, because the cusps of P1 molar germ begin to form while the development of root has not started yet, which is a good time point to observe tooth eruption and root development. We found that CS-*Clcn7* siRNA nanoparticles directly targeted on some tooth tissues or cells. The early responding tissues to *Clcn7* siRNA located in enamel and dentin, later in root and periodontal tissue. The local injection of CS-*Clcn7* siRNA eventually resulted in impacted or delayed tooth eruption as we expected. We also tested the effect of local injection of chitosan formulated siRNA of *Atp6v0a3*, a gene involved in osteopetrosis^17^, and found that *Atp6v0a3* siRNA may also target tooth cells and cause tooth malformation and root dysplasia after 17 injection days (Supplementary Fig. 4). Thus the local injection of chitosan formulated siRNA of certain genes around tooth germ enables us to test a local gene silence or knock out effect during tooth development and this method should be widely used in the future.

In order to identify the above tooth malformations caused by the effect of ClC-7 on osteoclasts or on tooth cells, the tooth germs infected with *Clcn7* shRNA viruses were transplanted under kidney capsule which provides an osteoclasts-poor environment. The malformations in the transplanted tooth germs infected with *Clcn7* shRNA viruses provided the direct evidence of ClC-7’s contribution to the tooth development through tooth cells. Meanwhile the milder changes in the transplanted tooth germs infected with *Clcn7* shRNA viruses comparing to that *in situ* CS-*Clcn7* siRNA injection further confirmed that the contribution of ClC-7 deficient osteoclasts to the abnormal tooth phenotypes *in situ*.

ClC-7 deficiency caused the lower expressed DSP protein level in dentin in our study. Enamel and dentin changes had also been found in other osteopetrosis mouse models, such as *Src(−/−)* mice^20^. Comparing to the dentin, less effect was found on enamel of *Src(−/−)* mouse, which quite similar to our results. Thus ClC-7 may affect enamel and dentin development by regulating various key molecules of ameloblasts and odontoblasts. We did not find the lowered *Dspp* mRNA level in MDPC-23 cell line, which might be because of the different responses of *Dspp* to lowered *Clcn7* level *in vivo* and *in vitro*, and the lower ΔCT value of *Dspp* in MDPC-23 cells.

A recent study in 3-week old ClC-7 mutant mice found that the molars did not form roots and the incisors were smaller than their age-matched controls. But the authors did not find obvious changes in the enamel of incisors and they regarded that ClC-7 deficiency might not significantly disrupt amelogenesis^5^. The different enamel’s reaction to ClC-7 deficiency in the reference^5^ and our data might be related with the position of investigated enamel. We observed enamel changes in molars, while Wen *et al.* studied the ultrastruture of enamel in incisors. The incisors and molars showed different root abnormalities in osteopetrosis patients with *CLCN7* mutations^2^ and *Clcn7* gene knockout mice^5^. On the other hand, the incisors were covered by enamel only in one side and the incisor growth is quite different from molar, especially during their root development. The deeper mechanism involved in the above different enamel changes under ClC-7 deficiency condition needs further exploring.

The deformed roots and abnormal periodontal tissues in ClC-7 deficient group switched our attentions to dental follicle cells. As the heterogeneous cell lineage, DFCs may differentiate into several types of cells, such as periodontal-type, cementoblastic-type and osteoblastic-type^21^, ^22^, ^23^, ^24^. The upregulations of osteoblast related genes such as *Alp, Opn*, and *Bsp* in DFCs with TDMM induction confirmed the ability of DFCs to differentiate into osteoblasts; however, the above changes could be inhibited by *Clcn7* gene deficiency. Thus ClC-7 might take part in the regulating of osteogeneic differentiation of DFCs.

One interesting finding is that the mRNA level of *Tgfb1* was downregulated in this osteogenic process after lowering the ClC-7 level. TGF-β1 has been reported to be involved in the regulation of tooth development^25^,^26^. The release and activation of TGF-β1 stimulates the synthesis of various extracellular matrix proteins and inhibits their degradation which possibly results in tissue fibrosis^27^,^28^. Our previous work found that the deficient ClC-5 and ClC-3 were related with the upregulation of TGF-β1^29^,^30^. Here again we found TGF-β1 also being involved in the regulation of ClC-7 but with different mechanisms.

The interaction between DFCs and osteoclasts has also been studied previously^23^. Early in eruption, the coronal part of dental follicle accumulates monocytes which may differentiate into osteoclasts^31^. TRAP positive monocytes were present in the dental follicle prior to the onset of eruption and then declined in number during eruption^23^,^32^. Here we found that DFCs/monocytes co-culture system could induce the monocytes to differentiate into osteoclasts. The co-cultures of human/rat DFCs and osteoclast precursors have been applied in contact or non-contact systems before^33^,^34^. Due to the inhibition function of OPG secreted by DFC, both systems turned out a lower efficiency of TRAP+ cell formation^33^,^34^ and fewer and shallow resorption pits^33^. Those TRAP+ cells in co-culture of DFC and monocytes only formed few and shallow pits in dentin slices, which might also be foreign body giant cells^35^. Our contact co-culture of mouse DFCs and monocytes confirmed the above findings. Meanwhile, we found that the lowered expression of ClC-7 in DFCs resulted in less TRAP positive multinuclear cells, less dentin resorption pits and smaller resorption area. Thus ClC-7 deficiency in DFCs might inhibit osteoclastgenesis.

RANK–RANKL–OPG signaling axis and downstream transcription factors play essential roles in the regulation of osteoclastogenesis^36^. DFCs are assumed to secret RANKL/M-CSF through a paracrine way, whilst OPG secreted by DFCs may inhibit osteoclastogenesis^8^,^37^. Consistent with the previous data^8^,^38^, we found the mRNA expression of *Rankl* and *Opg* in DFCs, and the lowered *Clcn7* level downregulated *Rankl* and upregulated *Opg*, which cause the inhibition of osteoclastogenesis. We suggest that ClC-7 is involved in the process of osteoclastogenesis through DFCs mediated RANK–RANKL–OPG signaling pathway. Meanwhile, we admitted that the mechanisms by which osteoclast precursors interact with DFCs and differentiate into osteoclasts *in vitro* and *in vivo* are more complicated than we expected, and our findings indicated that multiple factors might take part in the interaction between DFCs and osteoclast precursors under ClC-7 deficiency conditions.

In conclusion, our experiments demonstrate that ClC-7 deficiency affects tooth development and eruption in several pathways. The dysfunction of ClC-7 may directly influence the formation and calcification of enamel and dentin, or impair the differentiations of DFCs and DFCs mediated osteoclastogenesis through RANK–RANKL–OPG signaling pathway during tooth eruption. All the above changes contribute to the malformed teeth and altered tooth eruption.

## Materials and Methods

### Local delivery of CS-siRNA nanoparticles around tooth germ. 

#### siRNA duplex preparation

The small interfering RNA (siRNA) duplexes targeting the mouse *Clcn7* gene with or without Cy5.5 fluorescence were designed and synthesized by GenePharma (Shanghai, China) according to GenBank™ (NM_011930.3). The sequence of *Clcn7* siRNA is: sense, 5′- GAG GAG GAA AGA CGA AUC ATT -3′; antisense, 5′- UGA UUC GUC UUU CCU CCU CTT -3′. The sequence of *Atp6v0a3* siRNA is: sense, 5′- GAA GGA CCC UUC UCU GAG A -3′; antisense, 5′- UCU CAG AGA AGG GUC CUU C -3′. The control siRNA (sense, 5′-UUC UCC GAA CGU GUC ACG UTT-3′; antisense, 5′-ACG UGA CAC GUU CGG AGA ATT-3′) was provided by GenePharma (Shanghai, China).

#### Preparation of CS-siRNA nanoparticle solution

Chitosan (CS) (100-300kDa, 93.37% deacetylation) (MP Biomedicals, US) was purified following the previously reported methods^39^. The chitosan stock solution was prepared in acetate buffer (300 mM, pH 5.5) to a final concentration of 0.8 mg/mL, then 1mL chitosan solution was mixed with 20 μl 100 μM siRNA solutions and stored at −20 °C till to use.

#### Local delivery of CS-siRNA nanoparticles and *in vivo* fluorescence imaging

The newborn Balb/c mice (Experimental Animal Center of Fourth Military Medical University) were anesthetized through the inhalation of ether. The left and right maxillary first molar germ was chosen as the targeting sites of *Clcn7* siRNA, *Atp6v0a3* siRNA, negative siRNA, respectively. In the preliminary experiments, CS-Cy5.5-siRNA was used to test the suitable injection location and the dosages of injected nanoparticles. The distribution of fluorescence was observed by *in vivo* imaging system (IVIS Lumina II, USA). Then the non-fluorescence CS-siRNA nanoparticles solution were injected 1–2 mm below the eye toward the tooth germ direction using a microsyringe, and the depths of injection was 2.5 mm in newborn mice. For the long-term gene silencing experiments, two additional local CS-siRNA injections were added every two days till alveolar bones were too hard to perform injection. All procedures of animal work were approved by Fourth Military Medical University, and all experiments were performed in accordance with guidelines and regulations of Experimental Animal Center of Fourth Military Medical University.

### Tooth germs kidney transplantation

pGCSIL-GFP lentiviral vector (GeneChem, China) was used to generate shRNA against *Clcn7* gene with the same siRNA targeting sequences. The plasmids were co-transfected into HEK 293T cells with lentiviral packaging plasmids to generate *Clcn7* shRNA lentivirus (*Clcn7*-shRNA group) or negative shRNA lentivirus (negative-shRNA group). The mandibular first molar germs dissected from E13.5 Balb/c mice were infected with *Clcn7*-shRNA lentivirus (5000MOI/tooth germ) with the medium containing Dulbecco Modified Eagle Medium (DMEM), 10% fetal bovine serum (FBS, Gibco, USA) and 5 μg/ml polybrene (GeneChem, China). After 24 hours’ infection, the tooth germs were transferred to the transwell system, and the medium was switched to complete medium and cultured for about18 hours^40^,^41^. Then the tooth germs were transplanted into the renal capsule of adult male Balb/c mice. After 2 and 4 weeks of transplantation, micro CT scanning was performed to observe the tooth formation. The kidneys containing tooth germs were harvested for histological analysis and immunohistochemical staining after 4 weeks of transplantation.

### Micro-CT scanning and analysis

Siemens Inveon system (Siemens, Germany) was used for the live animal scanning and three dimension images reconstruction. For CS-siRNA nanoparticles injection model, the heads of P17 mice were separated and scanned with the parameters as 80kV, 500 μA, 500 ms exposure time, and scan angle of 360°. For the tooth germ and kidney subcapsular transplantation, after 2 weeks and 4 weeks of the transplantation, the mice and the kidneys with tooth germs were scanned with the same parameters as above, respectively. The experiment group and the controls were subjected to the same radiation.

### Histological analysis and immunocytochemical staining

All the tooth samples and the kidneys containing tooth germs were performed for hematoxylin & eosin staining and AZON staining, respectively. The sABC immunohistochemistry staining kit (Boster, Wuhan, China) was used for the immunohistochemical staining or immunocytochemical staining. Antibodies included mouse monoclonal IgG anti-DSP (kindly provided by Dr. Rong Zhang from Fourth Military Medical University), mouse monoclonal anti-vimentin antibody, mouse monoclonal anti-pan cytokeratin antibody (Abcam, UK).

### Cell Culture

#### Cell lines and primary culture of mouse dental follicle cells (DFCs)

Odontoblast MDPC-23 cell line (gift from Dr. C.T. Hanks and Dr. Jacques E. Nor, University of Michigan) and ameloblast LS8 cell line (kindly provided by Dr. Malcolm L. Snead in USC, Los Angeles, CA) were cultured in DMEM containing 10% FBS at 37 °C in a 5% CO_2_ humidified atmosphere^42^,^43^. Using modified Wise’s method^21^, the dental follicle tissues were dissected from the tooth germs of 3–5 postnatal days Balb/c mouse, cut into pieces and digested in a solution of 2 g/L type I collagenase for 1 hour at 37 °C. The cells were cultured in α-MEM containing 20% FBS, sodium pyruvate (0.1 g/l), streptomycin (100 μg/ml) and penicillin (100 U/ml). The 3^rd^ passage cells were used for all the experiments.

#### Lentivirus infection of cells or siRNA transfection

Cells (LS8 cells, MDPC-23 cell, and DFCs) grew up to 30% to 40% confluence were transfected with *Clcn7* siRNA or control siRNA using lipofectin 2000, or infected with *Clcn7* (or control) shRNA lentivivus at an MOI of 100. The mRNA were harvested after 72 hours for qRT-PCR analysis.

#### Quantitative realtime polymerase chain reaction (qRT-PCR)

Quantitative RCR was performed as previously described methods^39^. The specific primers for amplification of *Gapdh, Clcn7, M-csf* (macrophage colony stimulating factor), *Trap* (acid phosphatase 5, tartrate resistant), *Ctsk* (cathepsin K) and *Rank* (receptor activator of nuclear factor-κB), *Rankl* (receptor activator of nuclear factor-κB ligand), *Opg* (osteoprotegerin), *Alp* (alkaline phosphatase), *Bsp* (bone sialoprotein)*, Tgfb1*(transforming growth factor-β1), *Cap* (cementum attachment protein), *Opn* (osteopontin), and *Col1* (collagen type І) are shown in Supplementary Table 1.

### Osteogenic assay

Treated dentin matrix medium (TDMM) was prepared as the following methods: After being soaked in deionized water for 5 hours with an interval of 20 minutes ultrasonic treatment per hour, the cattle dentin matrices were serially soaked in 17% EDTA, 10% EDTA, and 5% EDTA for 5~10 minutes, with a deionized water wash for 10 minutes in an ultrasonic cleaner during each intervals. The treated dentin matrices were ground into powder with the treatment of liquid nitrogen, and sterilized with Co^60^ irradiation. TDMM was made with a ratio of 20 g dentin powder per 100 ml α-MEM medium and incubated for 5 days at 37 °C, then the medium were filtered using a 0.22  μm filter and stored at −20 °C. DFCs were treated with TDMM for 3 or 21 days, then performed with qRT-PCR or Alizarin red staining.

### Osteoclast function assay

#### Co-culture of DFCs and monocytes (MNCs)

The monocytes were isolated using a Mouse Bone Marrow Monocyte Cell Isolation Kit (TBD science, China) from the femur and tibia of 6-weeks-old male Balb/c mice according to the manufacturer’s protocol. For the establishment of DFCs/MNCs co-culture system, cell density of DFCs and MNCs were adjusted to 10^5^/ml and 10^6^/ml, respectively. LS8 cells/MNCs co-culture, DFCs alone, MNCs alone were set as the controls.

#### TRAP staining and dentin resorption assay

After being co-cultured for 7 days, the DFCs/MNCs were stained for TRAP using Acid Phosphatase, Leucocyte (TRAP) Kit (Sigma, USA). The number of TRAP-positive multinucleated cells (≥3 multinuclear) was counted and compared among *Clcn7*-shRNA group, negative-shRNA group and control group. After 14 days co-culture of DFCs/MNCs on dentin slices (4 mm × 4 mm × 200 μm), the number of resorptive lacunae of each dentine slice was counted under the scanning electron microscopy (SEM). The areas of the lacunas were measured with NIH Image J software (NIH, USA) for at least 50 lacunas each group.

### Statistical analysis

All the experiments were repeated at least three times and the data were presented as mean ± SD (standard deviation). Comparison between two different groups was performed using Independent-Samples T Test. Comparison among three different groups were performed using a One-Way ANOVO, and multiple comparisons were performed using LSD test and SNK test when data meet the homogeneity of variance, otherwise Dunnett T3 test and Dunnett C test were performed. The difference was considered to be statistically significant when the P value was <0.05 or 0.01.

## Additional Information

**How to cite this article**: Wang, H. *et al.* ClC-7 Deficiency Impairs Tooth Development and Eruption. *Sci. Rep.* **6**, 19971; doi: 10.1038/srep19971 (2016).

## Supplementary Material

Supplementary Information

## References

[b1] Pangrazio A. *et al.* Molecular and clinical heterogeneity in CLCN7-dependent osteopetrosis: report of 20 novel mutations. Hum Mutat. 31, E1071–E1080 (2010). 19953639 10.1002/humu.21167

[b2] Xue Y., Wang W., Mao T. & Duan X. Report of two Chinese patients suffering from CLCN7-related osteopetrosis and root dysplasia. J Craniomaxillofac. Surg. 40, 416–420 (2012). 21962762 10.1016/j.jcms.2011.07.014

[b3] Kornak U. *et al.* Loss of the ClC-7 chloride channel leads to osteopetrosis in mice and man. Cell 104, 205–215 (2001). 11207362 10.1016/s0092-8674(01)00206-9

[b4] Lu X. *et al.* A new osteopetrosis mutant mouse strain (ntl) with odontoma-like proliferations and lack of tooth roots. Eur. J Oral Sci. 117, 625–635 (2009). 20121924 10.1111/j.1600-0722.2009.00690.x PMC4066957

[b5] Wen X., Lacruz R. S. & Paine M. L. Dental and Cranial Pathologies in Mice Lacking the Cl(−) /H(+) -Exchanger ClC-7. Anat. Rec. (Hoboken.) 298, 1502–1508 (2015). 25663454 10.1002/ar.23118 PMC4503507

[b6] Kasper D. *et al.* Loss of the chloride channel ClC-7 leads to lysosomal storage disease and neurodegeneration. EMBO J 24, 1079–1091 (2005). 15706348 10.1038/sj.emboj.7600576 PMC554126

[b7] Helfrich M. H. Osteoclast diseases and dental abnormalities. Arch. Oral Biol 50, 115–122 (2005). 15721137 10.1016/j.archoralbio.2004.11.016

[b8] Wise G. E., Lumpkin S. J., Huang H. & Zhang Q. Osteoprotegerin and osteoclast differentiation factor in tooth eruption. J Dent. Res 79, 1937–1942 (2000). 11201042 10.1177/00220345000790120301

[b9] Weinert S. *et al.* Transport activity and presence of ClC-7/Ostm1 complex account for different cellular functions. EMBO Rep. 15, 784–791 (2014). 24820037 10.15252/embr.201438553 PMC4196982

[b10] Sartelet A. *et al.* A missense mutation accelerating the gating of the lysosomal Cl−/H+-exchanger ClC-7/Ostm1 causes osteopetrosis with gingival hamartomas in cattle. Dis Model. Mech. 7, 119–128 (2014). 24159188 10.1242/dmm.012500 PMC3882054

[b11] Neutzsky-Wulff A. V., Karsdal M. A. & Henriksen K. Characterization of the bone phenotype in ClC-7-deficient mice. Calcif. Tissue Int. 83, 425–437 (2008). 18958510 10.1007/s00223-008-9185-7

[b12] Neutzsky-Wulff A. V. *et al.* Severe developmental bone phenotype in ClC-7 deficient mice. Dev. Biol 344, 1001–1010 (2010). 20599900 10.1016/j.ydbio.2010.06.018

[b13] Duan X. Ion channels, channelopathies, and tooth formation. J Dent. Res 93, 117–125 (2014). 24076519 10.1177/0022034513507066

[b14] Hou J., Situ Z. & Duan X. ClC chloride channels in tooth germ and odontoblast-like MDPC-23 cells. Arch. Oral Biol 53, 874–878 (2008). 18466876 10.1016/j.archoralbio.2008.03.009

[b15] Kong Y. Y. *et al.* OPGL is a key regulator of osteoclastogenesis, lymphocyte development and lymph-node organogenesis. Nature 397, 315–323 (1999). 9950424 10.1038/16852

[b16] Ohgi K. *et al.* A novel inhibitory mechanism of nitrogen-containing bisphosphonate on the activity of Cl- extrusion in osteoclasts. Naunyn Schmiedebergs Arch. Pharmacol 386, 589–598 (2013). 23564016 10.1007/s00210-013-0857-0

[b17] Frattini A. *et al.* Defects in TCIRG1 subunit of the vacuolar proton pump are responsible for a subset of human autosomal recessive osteopetrosis. Nat Genet 25, 343–346 (2000). 10888887 10.1038/77131

[b18] Weinert S. *et al.* Lysosomal pathology and osteopetrosis upon loss of H+-driven lysosomal Cl− accumulation. Science 328, 1401–1403 (2010). 20430974 10.1126/science.1188072

[b19] Pan M., Ni J., He H., Gao S. & Duan X. New paradigms on siRNA local application. BMB. Rep. 48, 147–152 (2015). 25081998 10.5483/BMBRep.2015.48.3.089 PMC4453025

[b20] Koehne T. *et al.* Osteopetrosis, osteopetrorickets and hypophosphatemic rickets differentially affect dentin and enamel mineralization. Bone 53, 25–33 (2013). 23174213 10.1016/j.bone.2012.11.009

[b21] Handa K. *et al.* Progenitor cells from dental follicle are able to form cementum matrix *in vivo*. Connect. Tissue Res 43, 406–408 (2002). 12489190 10.1080/03008200290001023

[b22] Morsczeck C. *et al.* *In vitro* differentiation of human dental follicle cells with dexamethasone and insulin. Cell Biol Int. 29, 567–575 (2005). 15951208 10.1016/j.cellbi.2005.03.020

[b23] Wise G. E. & Fan W. Changes in the tartrate-resistant acid phosphatase cell population in dental follicles and bony crypts of rat molars during tooth eruption. J Dent. Res 68, 150–156 (1989). 2465331 10.1177/00220345890680021001

[b24] Wise G. E., Lin F. & Fan W. Culture and characterization of dental follicle cells from rat molars. Cell Tissue Res 267, 483–492 (1992). 1571962 10.1007/BF00319370

[b25] Chai Y. *et al.* Specific transforming growth factor-beta subtypes regulate embryonic mouse Meckel’s cartilage and tooth development. Dev. Biol 162, 85–103 (1994). 8125201 10.1006/dbio.1994.1069

[b26] Oka S. *et al.* Cell autonomous requirement for TGF-beta signaling during odontoblast differentiation and dentin matrix formation. Mech. Dev. 124, 409–415 (2007). 17449229 10.1016/j.mod.2007.02.003 PMC2704601

[b27] Branton M. H. & Kopp J. B. TGF-beta and fibrosis. Microbes. Infect. 1, 1349–1365 (1999). 10611762 10.1016/s1286-4579(99)00250-6

[b28] Border W. A. & Noble N. A. Transforming growth factor beta in tissue fibrosis. N. Engl. J Med 331, 1286–1292 (1994). 7935686 10.1056/NEJM199411103311907

[b29] Duan X. *et al.* ClC-5 regulates dentin development through TGF-beta1 pathway. Arch. Oral Biol 54, 1118–1124 (2009). 19878925 10.1016/j.archoralbio.2009.09.008

[b30] Wang H. *et al.* Chloride channel ClC-3 promotion of osteogenic differentiation through Runx2. J Cell Biochem. 111, 49–58 (2010). 20506205 10.1002/jcb.22658

[b31] Marks S. C. Jr. The basic and applied biology of tooth eruption. Connect. Tissue Res 32, 149–157 (1995). 7554911 10.3109/03008209509013718

[b32] Wise G. E., Marks S. C. Jr. & Cahill D. R. Ultrastructural features of the dental follicle associated with formation of the tooth eruption pathway in the dog. J Oral Pathol 14, 15–26 (1985). 3918150 10.1111/j.1600-0714.1985.tb00461.x

[b33] Sun H. *et al.* Regulation of OPG and RANKL expressed by human dental follicle cells in osteoclastogenesis. Cell Tissue Res 362, 399–405 (2015). 26149648 10.1007/s00441-015-2214-8

[b34] Wise G. E., Yao S., Zhang Q. & Ren Y. Inhibition of osteoclastogenesis by the secretion of osteoprotegerin *in vitro* by rat dental follicle cells and its implications for tooth eruption. Arch. Oral Biol 47, 247–254 (2002). 11839361 10.1016/s0003-9969(01)00109-1

[b35] Ten H. B. *et al.* The Foreign Body Giant Cell Cannot Resorb Bone, But Dissolves Hydroxyapatite Like Osteoclasts. PLoS One 10, e0139564 (2015). 26426806 10.1371/journal.pone.0139564 PMC4591016

[b36] Yasuda H. *et al.* Osteoclast differentiation factor is a ligand for osteoprotegerin/osteoclastogenesis-inhibitory factor and is identical to TRANCE/RANKL. Proc. Natl. Acad Sci. USA 95, 3597–3602 (1998). 9520411 10.1073/pnas.95.7.3597 PMC19881

[b37] Liu D., Yao S., Pan F. & Wise G. E. Chronology and regulation of gene expression of RANKL in the rat dental follicle. Eur. J Oral Sci. 113, 404–409 (2005). 16202028 10.1111/j.1600-0722.2005.00245.x

[b38] Suzuki T., Suda N. & Ohyama K. Osteoclastogenesis during mouse tooth germ development is mediated by receptor activator of NFKappa-B ligand (RANKL). J Bone Miner. Metab 22, 185–191 (2004). 15108059 10.1007/s00774-003-0481-z

[b39] Gao S. *et al.* Megalin-mediated specific uptake of chitosan/siRNA nanoparticles in mouse kidney proximal tubule epithelial cells enables AQP1 gene silencing. Theranostics. 4, 1039–1051 (2014). 25157280 10.7150/thno.7866 PMC4142293

[b40] Song Y. *et al.* Application of lentivirus-mediated RNAi in studying gene function in mammalian tooth development. Dev. Dyn. 235, 1334–1344 (2006). 16628661 10.1002/dvdy.20706

[b41] Song Y., Yan M., Muneoka K. & Chen Y. Mouse embryonic diastema region is an ideal site for the development of ectopically transplanted tooth germ. Dev. Dyn. 237, 411–416 (2008). 18213586 10.1002/dvdy.21427 PMC3010765

[b42] Duan X. *et al.* Odontoblast-like MDPC-23 cells function as odontoclasts with RANKL/M-CSF induction. Arch. Oral Biol 58, 272–278 (2013). 22770625 10.1016/j.archoralbio.2012.05.014

[b43] Yang T. *et al.* High amounts of fluoride induce apoptosis/cell death in matured ameloblast-like LS8 cells by downregulating Bcl-2. Arch. Oral Biol 58, 1165–1173 (2013). 23598055 10.1016/j.archoralbio.2013.03.016

