# Supplementary material for: ClC-7 Deficiency Impairs Tooth Development and Eruption
Source: Sci Rep. 2016 Feb 1;6:19971. doi: 10.1038/srep19971 (PMC4734291; doi:10.1038/srep19971)
Supplement: Supplementary Information [file srep19971-s1.doc]

**Supplementary Information**

**ClC-7 Deficiency Impairs Tooth Development and Eruption**

He Wang,^1,2#^ Meng Pan,^1,3#^ Jinwen Ni,^1#^ Yanli Zhang^1^, Yutao Zhang^1,3^, Shan Gao^4^, Jin Liu^1^ , Zhe Wang^1^, Rong Zhang^5^, Huiming He,^3*^ Buling Wu,^2*^ and Xiaohong Duan^1*^

^1^State Key Laboratory of Military Stomatology, Department of Oral Biology, Clinic of Oral Rare and Genetic Diseases, School of Stomatology, The Fourth Military Medical University, Xi’an, 710032, People’s Republic of China. ^2^Department of Stomatology, Nanfang Hospital, Guangzhou; College of Stomatology, Southern Medical University, Guangzhou, 510515, People’s Republic of China. ^3^State Key Laboratory of Military Stomatology, Department of Prosthodontics, School of Stomatology, The Fourth Military Medical University, Xi’an, 710032, People’s Republic of China. 4 The Interdisciplinary Nanoscience Center (iNANO) and Department of Molecular Biology and Genetics, Aarhus University, 8000 Aarhus C, Denmark; Xiangya Stomatological Hospital, Central South University, Changsha 410078, China 5 State Key Laboratory of Military Stomatology, Department of Endodontics, School of Stomatology, The Fourth Military Medical University, Xi’an, 710032, People’s Republic of China.

# contributes equally in this work

*Co-corresponding authors

Contact information:

Xiaohong Duan:

State Key Laboratory of Military Stomatology, Department of Oral Biology, Clinic of Oral Rare and Genetic Diseases, School of Stomatology, the Fourth Military Medical University, 145 West Changle Road, Xi’an 710032, P. R. China. Tel: 86-29-84776169；Fax: 86-29-84776169；E-mail: [xhduan@fmmu.edu.cn](mailto:xhduan@fmmu.edu.cn)

**Supplementary figures**

**
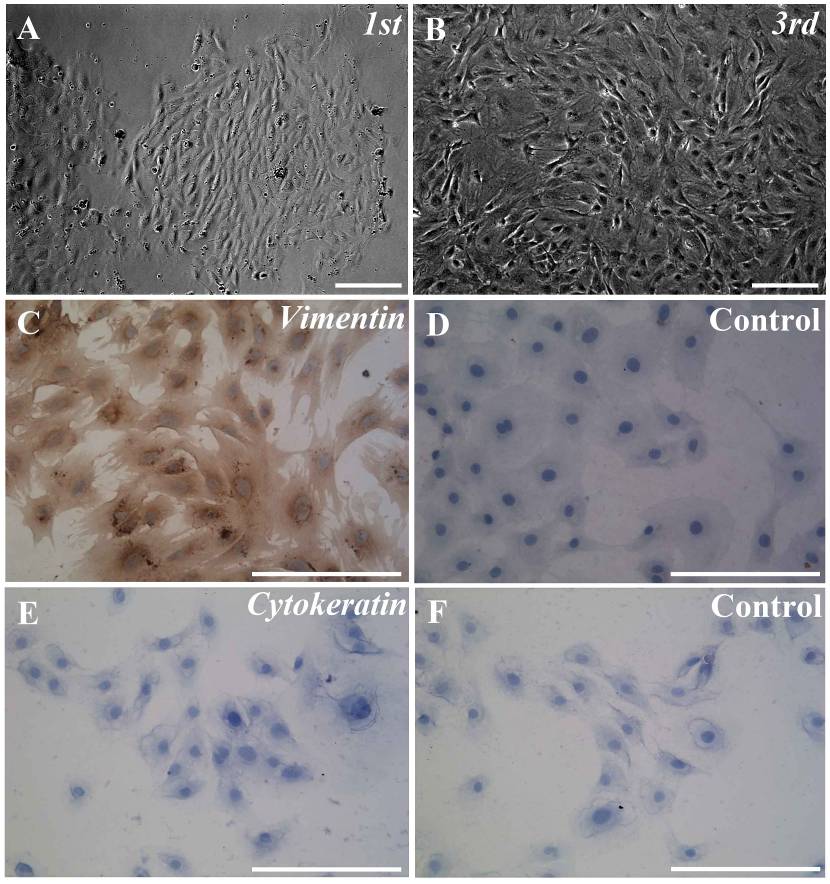
**

**Supplementary figure 1** **Characteristics of primarily cultured dental follicle cells (DFCs)**

A. Phase contrast images of DFCs in the first passage; B. Phase contrast images of DFCs in 3^rd^ passage. C and E. Immunoctochemistry staining of vimentin and cytokeratin in DFCs. D and F. Negative control for the immunoctochemistry staining of vimentin and cytokeratin in DFCs. Scale bars = 200μm.

**
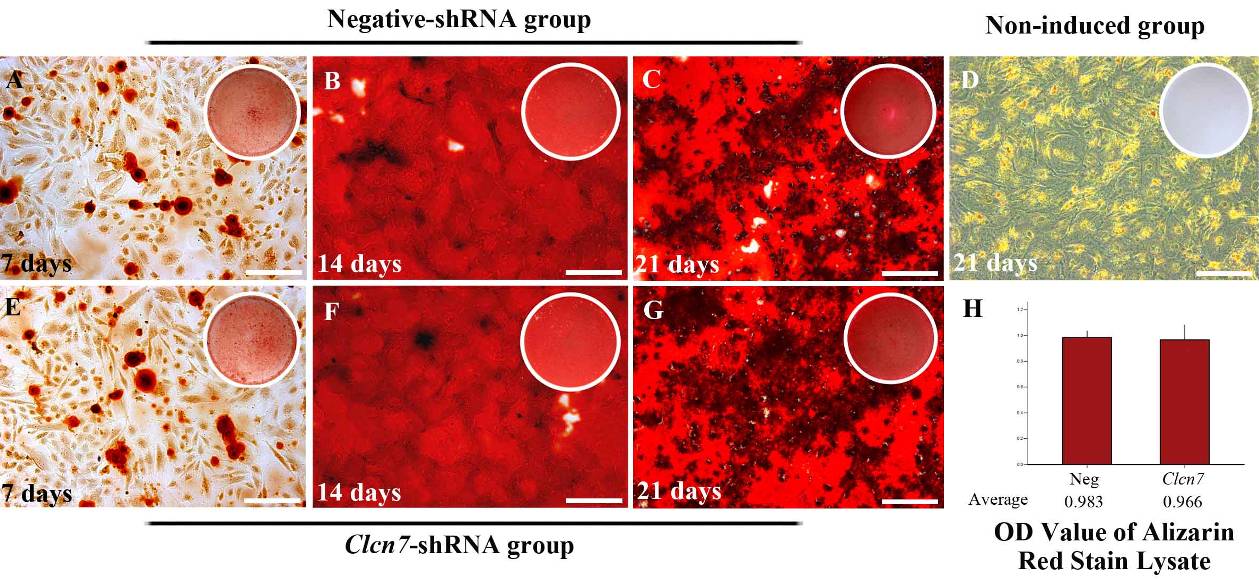
**

**Supplementary figure 2 ClC-7’s effect on the induced calcified ability of DFCs**

A~G. DFCs treated with osteogenic induction medium were stained by Alizarin red, then were photographed under a phase contrast microscope and Canon EF 100mm f/2.8 USM camera lens (top right). After osteogenic induction, DFCs gradually formed mineralized nodules at day 7, 14 and 21 (A~C). In contrast, the mineralized nodules were negligible in DFCs cultured in regular medium at day 21 (D). Scale bars = 200μm. There were not visible differences between negative-shRNA group (A~C) and *Clcn7-shRNA* group (E~G), OD value of alizarin stain lysate also showed the same negative result (H). ***P＜0.01*,**P＜0.05*. Data were presented as mean (SD).

**
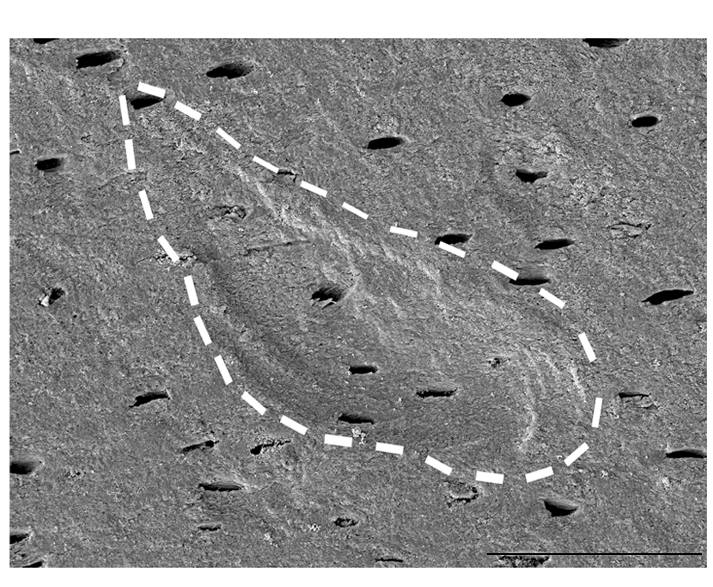
**

**Supplementary figure 3 SEM image of dentine resorption pit by osteoclasts**

After 14 days co-culture of DFCs/MNCs on dentin slices (4mm×4mm×200μm), resorptive lacunae in dentine slice was observed under the scanning electron microscopy (SEM). A representative small and shallow dentin resorption lacuna in the *Clcn7-*shRNA group was shown. Bars = 200μm.

**
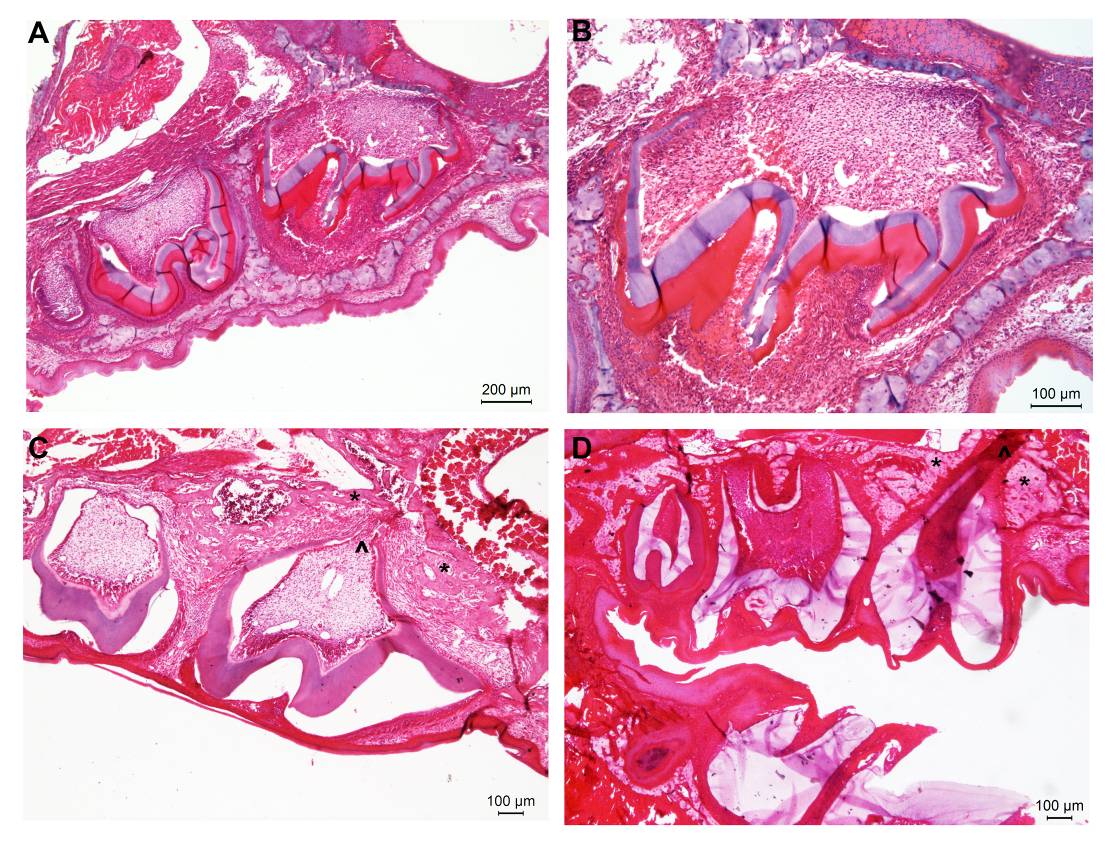
**

**Supplementary figure 4 H&E staining of molars treated with the local injection of CS- *Atp6v0a3*-siRNA particles**

The newborn Balb/c mice were anesthetized through the inhalation of ether. Chitosan formulated *Atp6v0a3* siRNA was injected 1-2 mm below the eye toward the first left upper molar direction using a microsyringe. Two additional local CS-siRNA injections were added every two days. The molars were harvested after 7 and 17 days and performed regular sectioning and H&E staining. A. On P7, the first molar treated with CS-*Atp6v0a3*-siRNA showed malformation of enamel. The integrity and continuity of enamel was interrupted. The tooth cusps became irregular. The second molar was almost intact. B is partial enlargement of figure A. C and D. On P17, the tooth cusps were deformed and the root formation were disrupted (^). More cortical bones were formed around roots (*). C and D are from two experiments. Scale bars = 200μm (A) and 100μm (B, C, D).

**Supplementary Table 1 – Primer sequences for real-time PCR**

| **Gene Name** | **Forward primer (5’-3’)** | **Reverse primer (5’-3’)** | **Accession number** | **Product length** |
| --- | --- | --- | --- | --- |
| ***Clcn7*** | GACAACAGCGAGAATCAGCTC | CCAATGAGGGCACAGATAACC | [NM_011930.3](http://www.ncbi.nlm.nih.gov/entrez/viewer.fcgi?db=nucleotide&id=141802324) | 104 |
| ***Rankl*** | CAGCATCGCTCTGTTCCTGTA | CTGCGTTTTCATGGAGTCTCA | [NM_011613.3](http://www.ncbi.nlm.nih.gov/entrez/viewer.fcgi?db=nucleotide&id=114842414) | 107 |
| ***Opg*** | ACCCAGAAACTGGTCATCAGC | CTGCAATACACACACTCATCACT | [NM_008764.3](http://www.ncbi.nlm.nih.gov/entrez/viewer.fcgi?db=nucleotide&id=113930715) | 157 |
| ***Dspp*** | ATTCCGGTTCCCCAGTTAGTA | CTGTTGCTAGTGGTGCTGTT | [NM_010080.2](http://www.ncbi.nlm.nih.gov/entrez/viewer.fcgi?db=nucleotide&id=111120321) | 128 |
| ***Enam*** | AAAACGGTGTCTTCCCTCCC | TTGGCTGACTTGGTCTCCAC | [NM_017468.3](http://www.ncbi.nlm.nih.gov/entrez/viewer.fcgi?db=nucleotide&id=226823204) | 87 |
| ***Alp*** | CCAACTCTTTTGTGCCAGAGA | GGCTACATTGGTGTTGAGCTTTT | [NM_001287172.1](http://www.ncbi.nlm.nih.gov/entrez/viewer.fcgi?db=nucleotide&id=563317854) | 110 |
| ***Bsp*** | CAGGGAGGCAGTGACTCTTC | AGTGTGGAAAGTGTGGCGTT | [NM_008318.3](http://www.ncbi.nlm.nih.gov/entrez/viewer.fcgi?db=nucleotide&id=297632386) | 158 |
| ***Tgfb1*** | CCACCTGCAAGACCATCGAC | CTGGCGAGCCTTAGTTTGGAC | [NM_011577.1](http://www.ncbi.nlm.nih.gov/entrez/viewer.fcgi?db=nucleotide&id=6755774) | 91 |
| ***Cap*** | GAGCGTGGTGCTTTTTCTGG | GCAAGGCGGCGTATATTGTG | [NM_013935.3](http://www.ncbi.nlm.nih.gov/entrez/viewer.fcgi?db=nucleotide&id=145279228) | 179 |
| ***Opn*** | TCTGATGAGACCGTCACTGC | AGGTCCTCATCTGTGGCATC | [NM_001204201.1](http://www.ncbi.nlm.nih.gov/entrez/viewer.fcgi?db=nucleotide&id=323668332) | 170 |
| ***Col1*** | TGACTGGAAGAGCGGAGAGT | GTTCGGGCTGATGTACCAGT | [NM_007742.3](http://www.ncbi.nlm.nih.gov/entrez/viewer.fcgi?db=nucleotide&id=118131144) | 151 |
| ***Gapdh*** | CATGTTCCAGTATGACTCCACTC | GGCCTCACCCCATTTGATGT | [NM_001289726.1](http://www.ncbi.nlm.nih.gov/entrez/viewer.fcgi?db=nucleotide&id=576080554) | 136 |
